# Isolation and Characterisation of Degradation Impurities in the Cefazolin Sodium Drug Substance

**DOI:** 10.3797/scipharm.1304-14

**Published:** 2013-06-04

**Authors:** Balasubramanian Sivakumar, Kannabiran Parthasarathy, Raman Murugan, Ramajeyabalan Jeyasudha, Saravanan Murugan, Rajendira Janardhan Saranghdar

**Affiliations:** Orchid Chemicals and Pharmaceuticals Limited, Research and Development Centre, Sozhanganallur, Chennai 600 119, Tamilnadu, India.

**Keywords:** Cefazolin sodium, Degradation Impurities, LC-MS/MS, NMR, β-Lactam

## Abstract

Two unknown impurities were detected in the cefazolin sodium bulk drug substance using gradient reversed-phase high-performance liquid chromategraphy (HPLC). These impurities were isolated by preparative HPLC and characterized by using spectroscopic techniques like LC-MS, LC-MS/MS, 1D, 2D NMR, and FT-IR. Based on the spectral data, the impurities have been characterized as *N*-(2,2-dihydroxyethyl)-2-(1*H*-tetrazol-1-yl)acetamide (Impurity-I) and 2-{carboxy[(1*H*-tetrazol-1-ylacetyl)amino]methyl}-5-methylidene-5,6-dihydro-2*H*-1,3-thiazine-4-carboxylic acid (Impurity-II). The structures of these impurities were also established unambiguously by co-injection into HPLC to confirm the retention time. To the best of our knowledge, these two impurities were not reported elsewhere.

## Introduction

Cephalosporin antibiotics are substitution products of 7-Aminocephalosporinic acid (7-ACA). Cefazolin sodium (CZS) is a first-generation cephalosporin antibiotic, has a tetrazolylacetyl side chain on the amino group and a 5-methyl-thiadiazolyl-thiomethyl group on the 3-position of 7-ACA. CZS is a broad-spectrum antibiotic, active *in vitro* against most Gram-positive and Gram-negative bacteria. Chemically, CZS is sodium (6*R*,7*R*)-3-{[(5-methyl-1,3,4-thiadiazol-2-yl)sulfanyl]methyl}-8-oxo-7-[(1*H*-tetrazol-1-yl-acetyl)amino]-5-thia-1-azabicyclo[4.2.0]oct-2-ene-2-carboxylate [1–4]. CZS is used in the form of intramuscular and intravenous injections for the treatment of infections of the respiratory system, urogenital system, infections of skin and soft tissues, biliary tract, bones, joints, and in endocarditis and septicaemia. CZS exhibits significant efficiency in the treatment of bacterial eye infections caused by staphylococci, pneumococci, and Gram-negative bacilli (*Escherichia coli*, *Klebsiella spp*., *Proteus spp*.) [5].

A literature search revealed that a number of high-performance liquid chromatographic (HPLC) methods have been developed to determine CZS in biological samples [6, 7]; CZS in buffered eye drops [8]; stress degradation [9]; stability of CZS sodium in polypropylene syringes and polyvinylchloride mini bags [10], and in heparinized and non-heparinized dialysis solution [11]. Photocatalytic degradation of CZS was reported along with the possible pathways of degradation [12]. However, the reported HPLC methods do not provide information about the unidentified impurities other than the ones listed in the European Pharmacopeia [13].

In CZS drug substances manufactured at Orchid Chemicals & Pharmaceuticals Ltd, two unknown impurities were observed at the relative retention time (RRT) of 0.08 and 0.20 by HPLC at a level of around 0.10%. These impurities were named as Impurity-I (RRT 0.08) and Impurity-II (RRT 0.20), found to be increasing to the level of 0.15% during stability studies. It is a mandatory requirement of regulatory authorities to identify and characterize any unknown impurity present or formed during stability studies with more than 0.1% in the drug substance and drug product [14–17]. A comprehensive study has been undertaken to isolate and characterize these two impurities by spectroscopic techniques [18–20]. This research paper describes the preparative HPLC isolation, identification, and characterization of these two unknown impurities of the CZS drug substance.

## Experimental

### Samples and Chemicals

The CZS sample manufactured at Orchid Chemicals and Pharmaceuticals Ltd, Alathur, Chennai, India was used for this study. Solvents were HPLC grade procured from Merck Specialities India Ltd. Potassium dihydrogen phosphate, sodium hydroxide, and orthophosphoric acid were HPLC grade supplied by Merck. Deuterated solvents for the NMR experiments were purchased from Euriso-top SA, France. The HPLC column, YMC Pack Pro, and preparative HPLC column YMC ODS-A were purchased from YMC, Japan and Thermo Electron Corporation. The water used for preparing the mobile phase was purified using the Millipore Milli-Q Plus (Milford, MA, USA) purification system.

### Analytical HPLC

The chromatographic separation was achieved on the YMC Pack Pro C18-RP column (250 X 4.6 mm and 5 μm as particle size) using the Waters Alliance 2695 model. The gradient LC method employed 6.8 gm of KH_2_PO_4_ in 1000 mL water (pH-adjusted to 6.80 with sodium hydroxide solution) as mobile phase A and a mixture of 500 mL of acetonitrile and 3.4 gm of KH_2_PO_4_ in 500 mL of water as mobile phase B. The LC gradient program was set as (T/%B) = 0/2, 7/15, 30/20, 35/20, 45/50, 50/50, and 55/2 with a post run-time of 10 min. The column temperature was maintained at 30 °C and the detection wavelength was set at 210 nm up to 4 minutes and after that 254 nm. The injection volume used was 20 μL and mobile phase A was used as a diluent.

### Forced Degradation of Cefazolin Sodium

The forced degradation study was performed on the CZS drug substance with the intention to ensure the degradation products’ separation from the analyte peak. Accordingly, a degradation study was conducted separately by treating CZS with acid, base, aqueous, peroxide, light, and heat.

#### Preparation of Sample Solution

About 125 mg of the CZS sample was dissolved in 50 mL of buffer (3.53 gm of potassium dihydrogen orthophosphate and 5.76 gm of anhydrous disodium hydrogen orthophosphate in 1000 mL of water) solution.

#### Acid Stressed Degradation

To 10 mL of the sample solution, 5 mL of 0.1N HCl solution was added and kept for about 2.5 hours at room temperature and then analysed by HPLC as per the method.

#### Base Stressed Degradation

To 10 mL of the sample solution, 10 mL of 0.2 N sodium hydroxide solution was added and analysed immediately by HPLC as per the method.

#### Peroxide Stressed Degradation

To 10 mL of the sample solution, 5 mL of 30% hydrogen peroxide solution was added and analysed immediately by HPLC as per the method.

#### Thermal Stressed Degradation

The sample solution was heated at 105°C for about 28 hours and analysed by HPLC as per the method.

#### Photolytic Degradation

The sample solution was exposed to UV radiation for about 33 hours and analyzed by HPLC.

#### Humidity Degradation

The sample solution was subjected to humidity degradation by keeping it at 25°C and 97% RH for about 34 hours and analyzed as per the HPLC method.

#### Results of the Forced Degradation Study

Impurity-I was observed at a level of 0.3% from acidic and photolytic degradation. The degradation conditions were optimised in such a way to increase Impurity–I to a level of 3%. The optimized condition was 1 gm of the CZS sample in 100 ml of water adjusted to a pH of 3.5 with orthophosphoric acid, and then exposed to UV light for about 12 hours.

Impurity-II was observed at a level of 2% from base degradation. The degradation condition was optimised to increase the Impurity-II content to 10% by heating the base degradation sample for about 10 minutes.

The other major impurities observed from stress degradation were 7-epimer of cefazolin, 5-methyl-1,3,4-thiadiazol-2-thiol (MMTD), cefazolin lactone, and cefazoloic acid. These impurities were listed in the European Pharmacopeia [13].

### Preparative HPLC

Isolation of the impurities was carried out using the Waters 2000 Prep HPLC equipped with a UV detector monitored at 210 nm and a YMC-ODS-A C18 (250 × 50 mm), 10 μm column was used. The impurities were eluted by using ratio of 99 parts water to 1 part acetonitrile at a flow rate of 30 mL/minute. The impurity fractions were collected from several injections and then pooled. These pooled fractions were concentrated separately by using the Rotavapor (Heidolph Laboratory 4002 control) under high vacuum. The aqueous solutions were subjected to lyophilization to obtain the impurity. The relative retention time of the isolated impurities were further confirmed by using HPLC spiking studies, and the molecular mass was confirmed by LC-MS. The purity of the isolated impurities was at around 90%.

### Liquid Chromatography Mass Spectrometry (LCMS)

LCMS experiments were carried out on a PE SCIEX API3000 LC-MS/MS equipped with a triple quadrupole mass analyzer and a TurboIonSpray sample introduction system. The chromatographic separation was achieved by the gradient HPLC systems Agilent 1100 G1311A(0) pump with Agilent 1100 G1329A(0) autosampler and Agilent 1100 DAD(0) detector. Analysis was carried out using the Hypersil ODS C18 (125 × 4 mm), 3μm column. Owing to the non-volatile nature of the phosphate-buffer mobile phase used in the HPLC related substances method, the chromatographic system was modified with 10 mM ammonium acetate, pH=7.5-adjusted with ammonia as mobile phase A and 100% acetonitrile was used as mobile phase B at a flow rate of 1.2 mL/min. The column oven temperature was maintained at 45 °C with an injection volume of 50μL with a 7 min injection delay. The UV detector was set to 254 nm. Gradient elution was performed by using mobile phase A and B. Gradient compositions were employed as (T/%B) = 0/2, 2/2, 4/15, 10/40, 11.5/65, 12 /65, 15/2, and 21/2. Mass analysis was performed in both positive and negative electrospray ionization modes. The capillary voltage was 5.5 kV. The interface temperature was 450°C. The impurity was subjected to a MS/MS study, wherein fragmentation of the various product ions was achieved by using different collision energies.

### NMR Spectroscopy

The ^1^H NMR, ^13^C NMR, and DEPT (Distortionless Enhancement by Polarisaton Transfer) experiments were performed with the Bruker Avance 400 MHz FT NMR spectrometer with a multinuclear BBO probe. DMSO-*d_6_* and D_2_O were used as solvents. The NMR spectra of Impurity-I are recorded in D_2_O, whereas the CZS and Impurity-II NMR spectra are recorded in DMSO-d_6_. The ^1^H chemical shift values were reported on the δ scale in ppm, relative to TMS/DSS (δ = 0.0 ppm) and in the ^13^C NMR, the chemical shift values were reported relative to DSS (δ = 0.0 ppm) for Impurity-I and DMSO-*d_6_* (δ = 39.50 ppm) as a reference in the case of Impurity-II. The DEPT spectra revealed the presence of methyl and methine carbons as positive peaks and methylene carbons as negative peaks. Homo- and heteronuclear chemical shift correlations were determined by the COSY and HSQC 2D NMR methods. Standard Bruker pulse sequences were applied by running Topspin 1.3 software. Typical samples were prepared for the ^13^C NMR experiments, which were 25 mg per 0.6 mL and 3–6 mg per 0.6 mL for ^1^H NMR measurements. The COSY and HSQC spectra were acquired in the magnitude and phase-sensitive mode. The HSQC spectrum was recorded with 64 scans, a relaxation delay of 1.5 s, a spectral width of 5341 and 16667 Hz in both dimensions, and 256 increments in t1 and 1K data points in t2.

### IR & UV Spectroscopy

The IR spectrum was recorded in the solid state as a KBr dispersion medium using the FT-IR (Perkin Elmer, Spectrum 65 & JASCO-FT-IR-430) spectrophotometer. The UV spectrum was recorded on a Shimadzu UV-Visible spectrophotometer 2550 using water as a medium.

## Results and Discussion

### Detection of Impurity-I and II

**Fig. 1:**
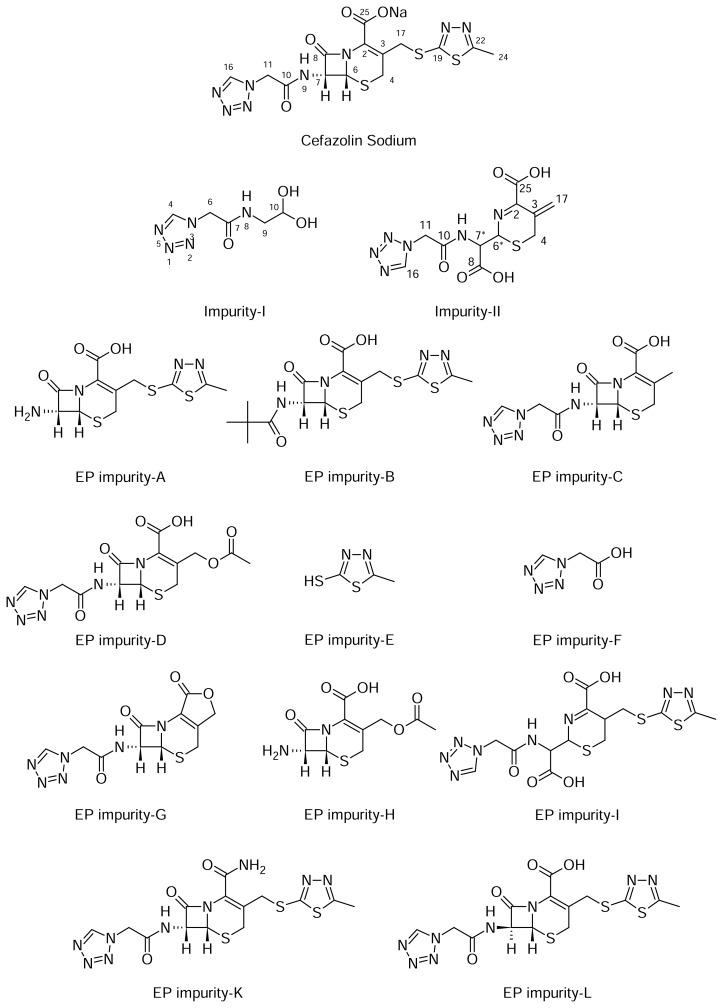
Structures of Cefazolin Sodium, Impurities-I+II, and other known Impurities

**Fig. 2:**
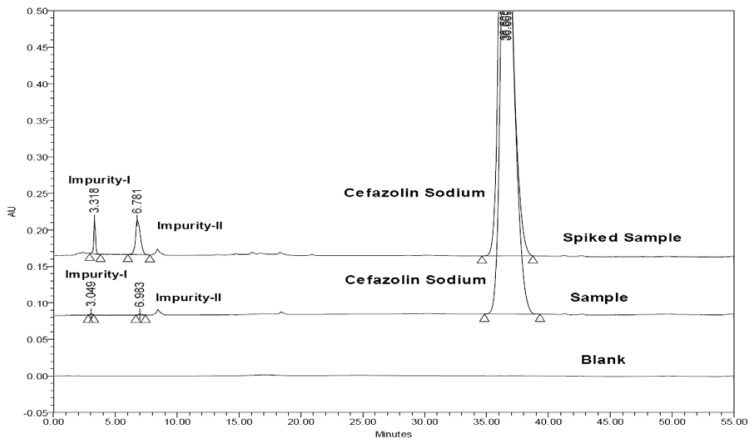
Overlaid chromatograms of blank and spiked impurities along with sample

The structures of Impurity-I, Impurity-II, and CZS are shown in Figure 1 along with the other known isolated/synthesized impurities, EP impurity-A, EP impurity-B, EP impurity-C, EP impurity-D, EP impurity-E, EP impurity-F, EP impurity-G, EP impurity-H, EP impurity-I, EP impurity-K, and EP impurity-L. A typical analytical HPLC overlaid chromatogram of the CZS sample and Impurities-I and -II spiked with the sample and the blank were analyzed using the method as described and shown in Figure 2. The target impurities under study were marked as Impurity-I and Impurity-II. The Relative Retention Times (RRT) of Impurity-I and -II are 0.084, and 0.202, respectively. These retention times indicate that Impurity-I and Impurity-II are more polar in nature than CZS.

### Characterization of Impurity-I

**Fig. 3:**
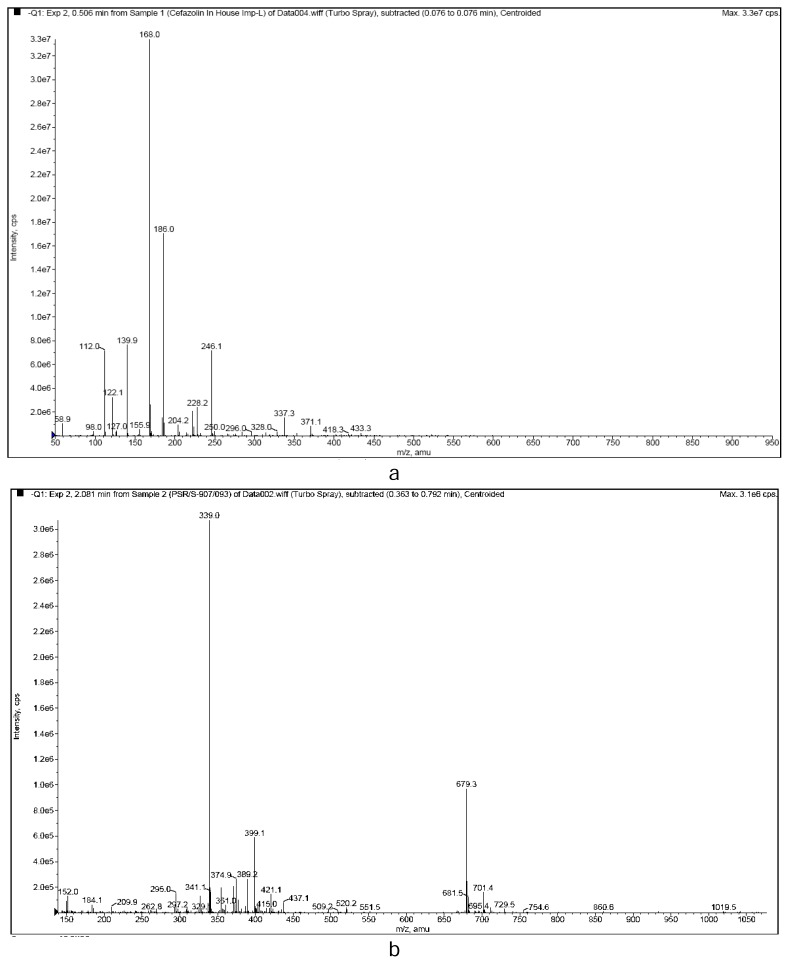
Mass Spectra of (a) Impurity-I and (b) Impurity-II

**Fig. 4:**
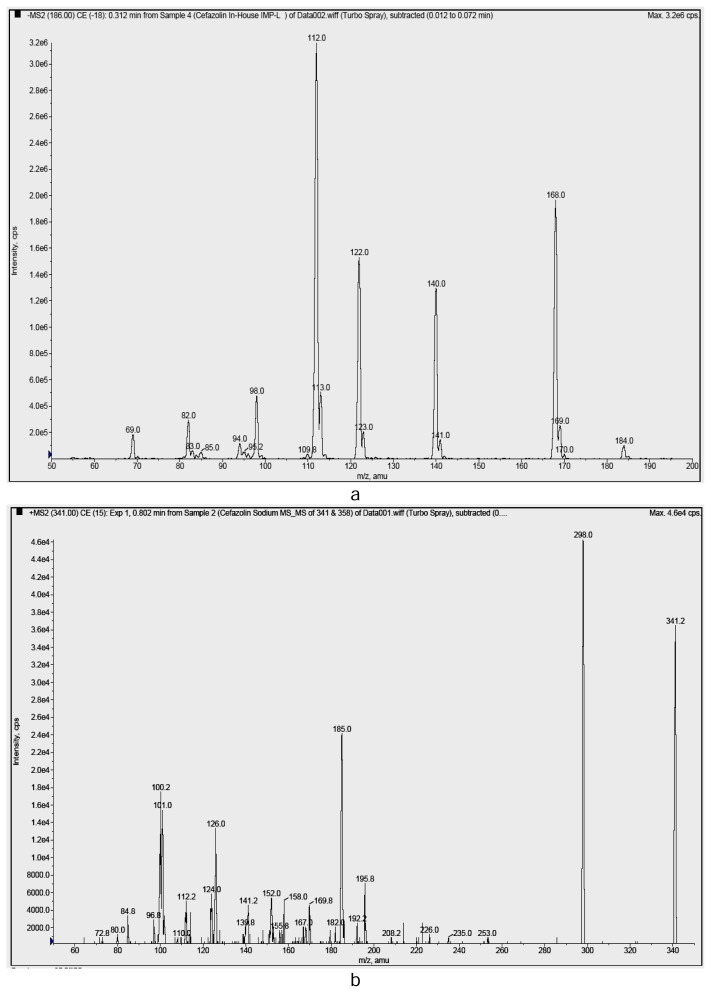
MS/MS spectra of (a) Impurity-I and (b) Impurity-II

The ESI mass spectrum of Impurity-I (Figure 3a) exhibits two distinct ions, m/z at 186 [M-H]^−^ and m/z 168 ([M-H-H_2_O]^−^. The molecular mass of 187 supports the absence of the cephem nucleus in the impurity. The major product ions (Figure 4a) in MS/MS were observed at m/z 168, 140, and 112.

**Fig. 5:**
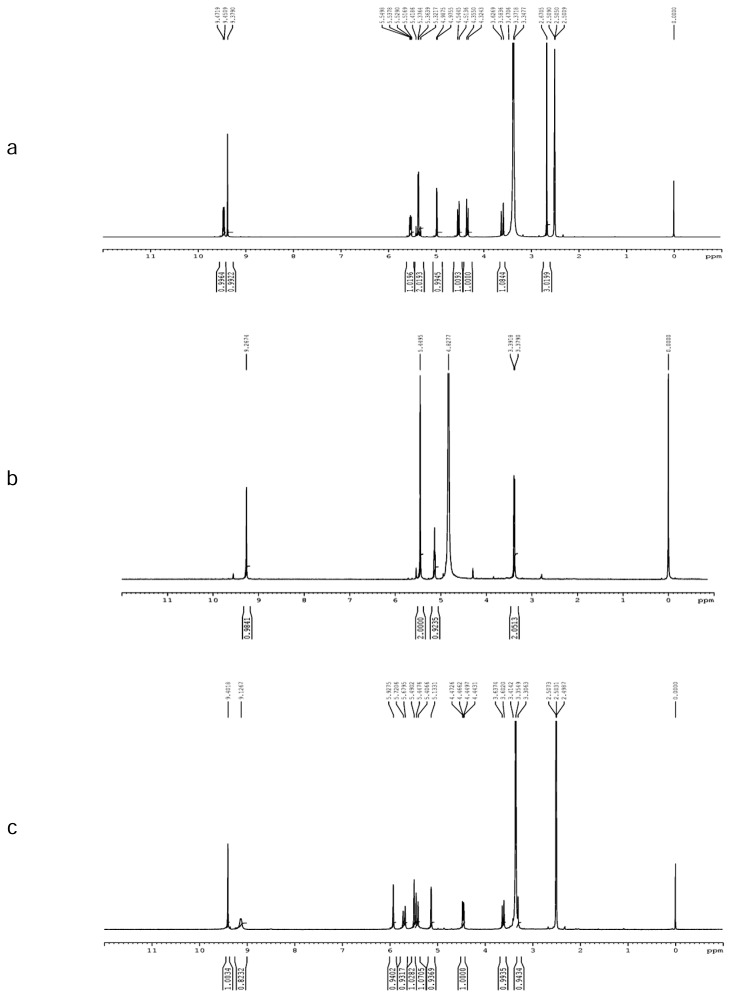
^1^H NMR spectra of (a) Cefazolin Sodium (b) Impurity-I and (c) Impurity-II

**Fig. 6:**
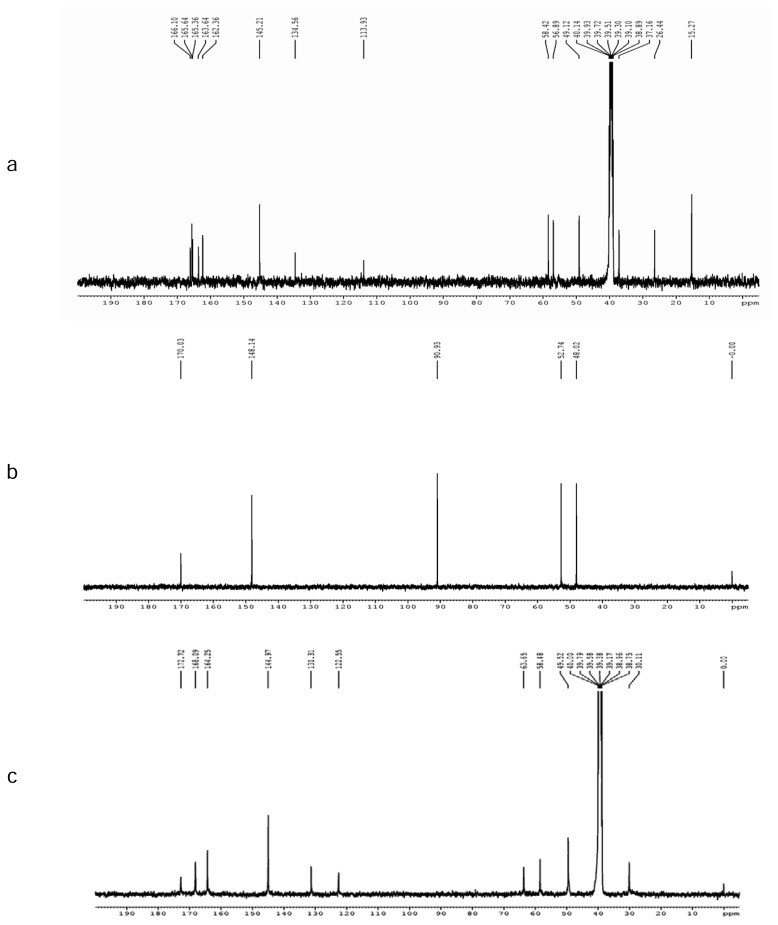
^13^C NMR spectra of (a) Cefazolin Sodium; (b) Impurity-I; and (c) Impurity-II

The ^1^H NMR and ^13^C NMR spectra of CZS and Impurity-I are shown in Figures 5(a), 6(a), and Figure 5(b), 6(b), respectively. The NMR and mass spectroscopic data of the isolated impurity was compared with those of the CZS data. In the ^1^H NMR spectrum of Impurity-I, hydrogen belongs to the cephem nucleus and the methylmercaptothiadiazole moiety at C-3 was not observed, and the methylene hydrogens and a methine hydrogen were newly observed while the hydrogen for the tetrazole was present at δ 9.25 ppm. The methylene group attached to tetrazole appeared as a singlet at δ 5.44 ppm (in CZS, the corresponding tetrazole and methylene hydrogen signals were observed at δ 9.15 and 5.42 ppm, respectively). The appearance of a characteristic doublet and triplet at δ 3.37 and 5.13 ppm, respectively, with a coupling constant J_1,3_ = 5.26 Hz indicate the presence of a –CH_2_-CH-moiety. A triplet at δ 5.13 ppm shows that the -CH- is attached to a more electronegative atom like oxygen.

In the ^13^C NMR spectrum, the signals appear at δ 145.6 and 50.2 ppm due to the tetrazole hydrogen-bearing carbon and the attached methylene carbon (in CZS, the corresponding carbon signals were observed at δ 145.7 and 50.1 ppm). A signal appeared at δ 170.0 ppm indicating the presence of a carbonyl group. The deshielding of the methine carbon (δ 90.0 ppm) indicates the attachment of two hydroxyl groups to that carbon. The DEPT results indicate that Impurity-I has two methylene carbons and two methine carbons.

The IR spectrum shows the characteristic stretching band at 3364 cm^−1^ due to the NH stretching vibration and 1689 cm^−1^ & 1552 cm^−1^ due to the Amide-I and Amide-II bond, respectively. One broad band around 3300 cm^−1^ indicates the presence of a hydroxyl group. No β-lactam carbonyl stretching was observed in the IR spectrum. The UV spectrum of the impurity shows the λ_max_ at 210 nm.

From the above NMR, MS, and FT-IR spectral results, the structure of Impurity-I was assigned as *N*-(2,2-dihydroxyethyl)-2-(1*H*-tetrazol-1-yl)acetamide having the molecular formula C_5_H_9_N_5_O_3_, an aldehyde hydrate derivative involving the 7-acyl moiety of CZS and tetrazole acetic acid.

An LCMS/MS study was performed in negative ionization mode. The major fragment ions at m/z 168, 140, 112 & 69 are showed in Figure 4(a). The product ion at m/z 168 corresponds to the dehydrated product of Impurity-I. The elimination of carbon monoxide followed by rearrangement yields the base peak at m/z 112. The fragment ion at m/z 69 was due to the tetrazol-1-ide moiety. These mass spectral fragments further confirm the elucidated structure.

The degradation pathway for the formation of the impurity is shown in Figure 9(a). Upon acid hydrolysis, cefazolin degraded to cefazoloic acid and subsequently formed lactone, then was decarboxylated with resulting bond migration, and expelled the cephem nuclues to give Impurity-I. A similar mechanism was reported in the literature for one of the cephalosporin drug substances, cefdinir [21, 22]. The ^1^H and ^13^C NMR signal assignments of CZS and Impurities-I are shown in Table 1.

### Characterization of Impurity-II

The ESI mass spectrum of Impurity-II (Figure 3(b)) shows a deprotonated molecular ion peak at m/z 339, which is 137 amu less than that of CZS, which indicates the absence of the MMTD moiety in the molecule. The distinct ions at m/z 399 and m/z 679 are due to [M+CH_3_COOH-H]^−^ and [2M-H]^−^. The MS/MS fragmentation pattern is shown in Figure 4(b). The major product ions were observed at m/z 298, 185, and 100.

**Fig. 7:**
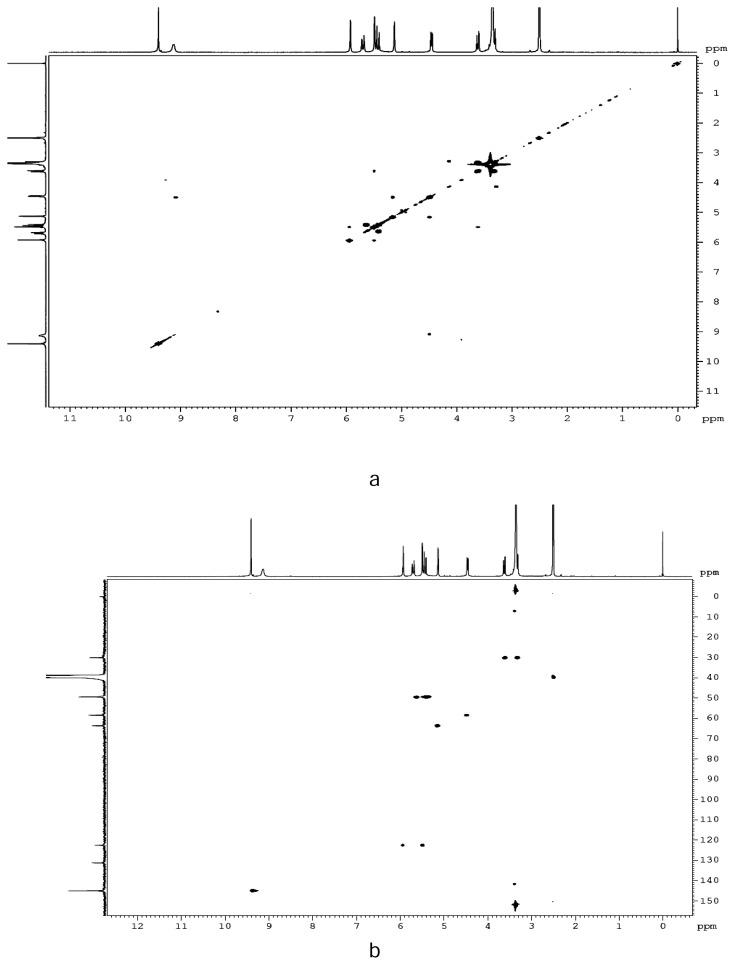
(a) HSQC and (b) COSY Spectrum of Impurity-II

The ^1^H NMR and ^13^C NMR spectra of Impurity-II are shown in Figure 5(c) and 6(c), respectively. From the ^1^H NMR spectrum, it is observed that two singlets at δ 5.49 ppm and δ 5.93 ppm correspond to two geminal olefinic protons, which is further confirmed by the intense off-diagonal contours in the ^1^H-^1^H COSY data (Figure 7(a)). A comparison of the ^1^H NMR spectra of the impurity and CZS shows an upfield shift for the 7-CH proton suggests the cleavage of the β-lactam ring. Also, the absence of the methyl signal in the aliphatic region in ^1^H/^13^C NMR data eliminates the presence of the methylmercapto-thiadiazole (MMTD) moiety at C-3 position of the cephem nucleus in the molecule. The DEPT spectrum shows the presence of three methylene carbons. The negative signal at δ 122.6 ppm ascertains that the olefenic carbon was present in the molecule. The HSQC data (Figure 7(b)) showed an intense contour between the hydrogen signals at δ 5.49 & 5.93 ppm and the corresponding carbon signal at δ 122.6 ppm, which confirms the presence of the =CH_2_ moiety in the molecule. The ^1^H and ^13^C NMR results indicate that there is no considerable change in the chemical shifts of hydrogen/carbon of the acyl substituent at C-7 position, which confirms that the tetrazole acetic acid moiety is present in Impurity-II. The FT-IR spectral data of Impurity-II show the absence of the band at ~1776 cm^−1^, which confirmed the cleavage of the β-lactam ring which was present in the FT-IR spectra of CZS. The UV spectrum of the impurity shows the λ_max_ at 226 nm, which matches that of the degradation impurity having =CH_2_ at C-3 position [24].

**Fig. 8:**
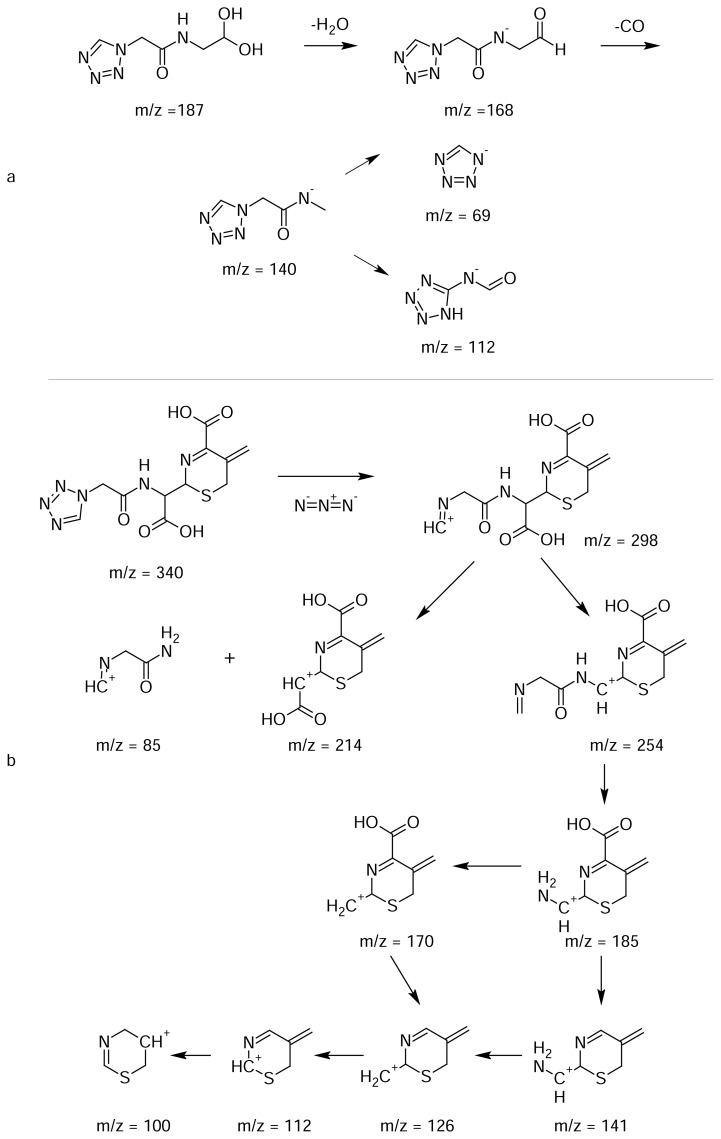
LC-MS/MS Fragmentation pattern of (a) Impurity-I and (b) Impurity-II

Based on the above spectroscopic results, the structure of Impurity-II was elucidated as 2-{carboxy[(1*H*-tetrazol-1-ylacetyl)amino]methyl}-5-methylidene-5,6-dihydro-2*H*-1,3-thiazine-4-carboxylic acid. This structure was well-supported by the MS/MS fragmentation pattern as shown in Figure 8(b). The ^1^H and ^13^C NMR signal assignments are shown in Table 1.

An LCMS/MS study was performed in positive ionization mode. The major fragment ions at m/z 298, 185 & 100 are shown in Figure 4(b). The expulsion of the azide moiety from the tetrazolyl side chain yielded the daughter ion at m/z 298. Decarboxylation followed by elimination of the methylidine amino acetaldehyde moiety resulted in the fragment ion at m/z 185. Successive elimination and rearrangement resulted in the fragment ion at m/z 100. These MS/MS fragmentation data further confirm the elucidated structure.

**Fig. 9:**
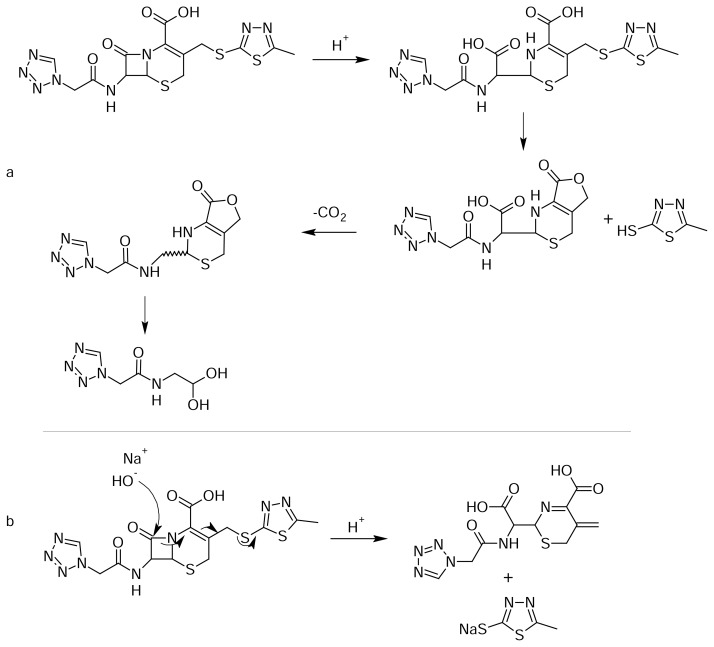
Degradation pathway for (a) Impurity-I and (b) Impurity-II

The degradation pathway for the formation of the impurity is also shown in Figure 9(b). Upon base degradation, the hydroxyl group attackeds the carbonyl carbon of the β-lactam ring. Subsequent bond migration expelled the MMTD moiety and resulted in the formation of the impurity. Similar compounds and their ^1^H NMR and UV absorption spectra were reported earlier as products of aminolysis and enzymatic hydrolysis of the cephalosporins [24, 25].

## Conclusion

Two of the degradation impurities of cefazolin sodium were detected in the HPLC analysis. These impurities were isolated by preparative HPLC and their structures were elucidated using the 1D NMR, 2D NMR, LC-MS, LC-MS/MS, and FT-IR spectral techniques. The LC-MS/MS fragmentation pattern and a possible degradation pathway were reported.

## Figures and Tables

**Tab. 1:** ^1^H and ^13^C NMR assignment for Cefzolin Sodium, Impurity-I and II

Atom	Cefazolin sodium				In-house Impurity-I				In-house Impurity-II			
	
	^1^H	ppm/J	^13^C	DEPT	^1^H	ppm/J	^13^C	DEPT	^1^H	ppm/J	^13^C	DEPT
2			134.6								168.1	
3			113.9								131.3	
4	2	*3.37&3.57/2d, 17.3	26.5	CH_2_	1	9.26/s	148.1	CH	2	3.31&3.60/2d, 14.2	30.1	CH_2_
6	1	4.98/d, 4.8	56.9	CH	2	5.44/s	52.7	CH2	1	5.13/s	63.7	CH
7	1	5.53/dd, 8.4, 4.8	58.4	CH			170		1	4.44/dd, 9.2, 2.6	58.5	CH
8			165.4								172.7	
9	NH	9.45/d, 8.4			2	3.37/d, 5.3	48	CH_2_	NH	9.13/bs		
10			165.6		1	5.12/t, 5.3	90.9	CH			164.3	
11	2	5.32&5.38,2d, 16.9	49.1	CH_2_					2	5.41&5.68/2d, 16.4	49.5	CH_2_
16	1	9.38/s	145.2	CH					1	9.40/s	144.9	CH
17	2	4.32&4.51/2d, 12.3	37.2	CH_2_					2	5.49&5.93/2s	122.6	CH_2_
19			163.6									
22			166.1									
24	3	2.67/s	15.3	CH_3_								
25			162.4								172.7	
